# p-Chloranilin – Ableitung eines BAR: Beurteilungswerte in biologischem Material

**DOI:** 10.34865/bb10647d11_2or

**Published:** 2026-06-30

**Authors:** Isabell Schönrath, Gabriele Leng, Hans Drexler, Andrea Hartwig, MAK Commission

**Affiliations:** 1 Currenta GmbH & Co. OHG, CUR-SIT-ABG-GS-BLM – Institut für Biomonitoring 51368 Leverkusen Deutschland; 2 40699 Erkrath Deutschland; 3 Friedrich-Alexander-Universität Erlangen-Nürnberg, Institut und Poliklinik für Arbeits-, Sozial- und Umweltmedizin Henkestraße 9–11 91054 Erlangen Deutschland; 4 Institut für Angewandte Biowissenschaften, Abteilung Lebensmittelchemie und Toxikologie, Karlsruher Institut für Technologie (KIT) Adenauerring 20a, Geb. 50.41 76131 Karlsruhe Deutschland; 5 Ständige Senatskommission zur Prüfung gesundheitsschädlicher Arbeitsstoffe, Deutsche Forschungsgemeinschaft, Kennedyallee 40, 53175 Bonn, Deutschland. Weitere Informationen: Ständige Senatskommission zur Prüfung gesundheitsschädlicher Arbeitsstoffe | DFG

**Keywords:** p-Chloranilin, Biologischer Arbeitsstoff-Referenzwert, BAR, Hintergrundbelastung

## Abstract

The German Commission for the Investigation of Health Hazards of Chemical Compounds in the Work Area evaluated the data for 4-chloroaniline [106-47-8] to derive a biological reference value (BAR). p-Chloroaniline is an intermediate in organic synthesis and is used in the manufacture of pharmaceuticals, plant protection products and dyes. As a potent inducer of methaemoglobin formation, the hazards regarding its acute toxicity surpass the carcinogenic potential. Hence, several accidents with a consecutive cyanosis are reported. Besides the methaemoglobin level, two other, more specific biomarkers are known: p-chloroaniline set free from the haemoglobin adduct and p-chloroaniline in urine. p-Chloroaniline after hydrolysis represents the sum of all *N*-conjugated phase II metabolites which are then back-converted to their mother compound. Based on studies of the general population, a BAR of 1 µg p-chloroaniline (after hydrolysis)/l urine has been derived. The smoking status did not influence the urinary levels of p-chloroaniline.

**Table:** 

**BAR (2025)**	**1 µg p-Chloranilin (nach Hydrolyse)/l Urin** Probenahmezeitpunkt: Expositions- bzw. Schichtende

**MAK-Wert**	**–**
Hautresorption (1990)	H
Sensibilisierende Wirkung (2008)	Sh
Krebserzeugende Wirkung (1990)	Kategorie 2
	
Synonyma	1-Amino-4-chlorbenzol
CAS-Nr.	106-47-8
Formel	C_6_H_6_ClN
Molmasse	127,57 g/mol
Schmelzpunkt	70 °C (IFA 2025)
Siedepunkt	232 °C (IFA 2025)
Relative Dichte bei 20 °C	1,43 g/cm^3^ (IFA 2025)
log K_OW_	1,83 (IFA 2025)

p-Chloranilin ist ein Intermediat in der organischen Synthese und wird zur Herstellung von Pharmazeutika, Pflanzenschutzmitteln und Farbstoffen verwendet.

## Metabolismus und Toxikokinetik

### Aufnahme, Verteilung und Elimination

p-Chloranilin kann inhalativ und dermal aufgenommen werden (Henschler 1990). Dabei kann der Aufnahmeweg über die Haut, wie in den im Folgenden diskutierten Unfällen geschildert (Herber 2024; Messmer et al. 2015; Pizon et al. 2009), zu signifikanten Methämoglobinkonzentrationen beim Menschen führen. Aufgrund der Hautpenetration wurde p-Chloranilin mit einem „H“ (Henschler 1990) und aufgrund positiver Befunde an Meerschweinchen sowie an der Maus im Hinblick auf eine kontaktsensibilisierende Wirkung mit „Sh“ gekennzeichnet (Hartwig 2009).

### Metabolismus

**Abb. 1:**
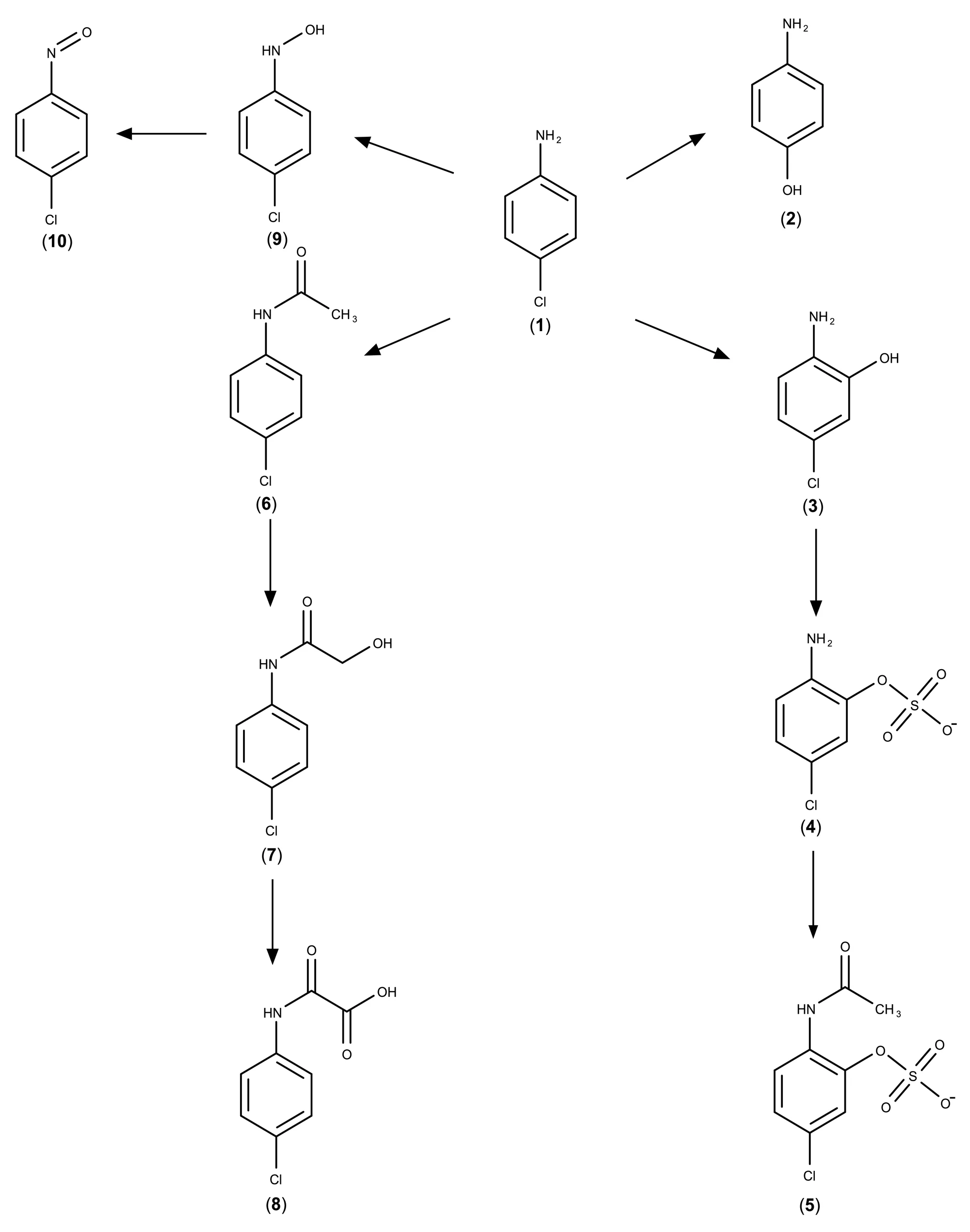
Metabolismus von p-Chloranilin, adaptiert nach Concise International Chemical Assessment Document 48: 4-Chloroaniline (WHO 2003). Die WHO übernimmt keine Verantwortung für den Inhalt oder die Richtigkeit dieser Bearbeitung.

Der Metabolismus von p-Chloranilin wurde bei Versuchstieren untersucht, verläuft beim Menschen ähnlich und konnte im Rahmen akuter Vergiftungsfälle beim Menschen bestätigt werden (WHO 2003). Nach der Aufnahme wird p-Chlor­anilin in der Leber zu ring- und *N*-hydroxylierten Verbindungen und ihren Konjugaten metabolisiert. Die vier Metabolismuspfade sind in Abbildung 1 dargestellt. Der aromatische Ring kann entweder an der para-Position unter Austausch des Chloridions (2) oder an der ortho-Position (3) hydroxyliert werden. Im letzteren Fall wird die Hydroxylgruppe im weiteren Verlauf mit einem Sulfat-Anion konjugiert (4) und die Aminogruppe acetyliert (5). Nach der *N*-Acetylierung von p-Chloranilin entsteht 4-Chloracetanilid (6). Wie schnell dieser Schritt im Menschen verläuft, hängt vom Acetyliererstatus der Person ab. Aus 4-Chloracetanilid (6) wird in einer weiteren Reaktion 4-Chlorglykolsäureanilid (7), das weiter zum Oxamsäure-Derivat (8) oxidiert wird. Die besonders reaktive Verbindung 4-Chlornitrosobenzol (10) entsteht, wenn p-Chloranilin an der Aminogruppe hydroxyliert (9) und im weiteren Verlauf oxidiert wird. Während die Metabolite (2), (5) und (8) im Urin nachgewiesen wurden, reagiert die Nitroso-Spezies (10) mit dem Hämoglobin und ist somit auch für die Methämoglobinbildung verantwortlich. Weitere Informationen zum Metabolismus und zur Toxikokinetik finden sich in der Dokumentation der WHO (2003).

(1) p-Chloranilin, (2) 4-Hydroxyanilin, (3) 2-Hydroxy-4-chloranilin, (4) 2-Amino-5-chlorphenylsulfat, (5) *N*-Acetyl-2-amino-5-chlorphenylsulfat, (6) 4-Chlor-*N*-acetanilid, (7) 4-Chlorglykolanilid, (8) *N*-(4-Chlorphenyl)oxamsäure, (9) 4-Chlor-*N*-hydroxyanilin, (10) 4-Chlornitrosobenzol.

## Kritische Toxizität

Im Vergleich zum Anilin ist p-Chloranilin mit einem Hämoglobin-Bindungsindex von 569 um den Faktor 25 stärker wirksam als Anilin mit einem Hämoglobin-Bindungsindex von 22 (Sabbioni 1994). Bei akuten Vergiftungen kommt es zu einer Zyanose (Herber 2024). Neben akuten Vergiftungen bei der Arbeit (Herber 2024; Messmer et al. 2015; Pizon et al. 2009) wurde auch schon über Frühgeborene mit schwerer Methämoglobinämie berichtet, deren Brutkästen versehentlich mit Chlorhexidin-Lösung statt destilliertem Wasser gereinigt wurden. Chlorhexidin kann spontan zu p-Chloranilin zerfallen (van der Vorst et al. 1990). Die Methämoglobinbildung wird als Ursache für die kanzerogene Wirkung von p-Chloranilin diskutiert, da durch den Abbau der nicht mehr intakten Erythrozyten die Milz geschädigt wird. Weitere Informationen zur Toxizität können der MAK-Begründung entnommen werden (Henschler 1990).

## Belastung und Beanspruchung

Aus akzidentiellen Belastungen wurden Korrelationen zwischen p-Chloranilin im Urin bzw. dem Hämoglobin-Addukt und dem gebildeten Methämoglobin als gesundheitlichem Effektmarker abgeleitet. Allerdings ist die genaue Expositionshöhe gegen p-Chloranilin bei diesen Studien unbekannt. Die Arbeit von Lewalter und Korallus (1985) zeigt den exakten zeitlichen Verlauf des Methämoglobingehalts im Blut sowie der gemessenen Konzentrationen von p-Chloranilin im Urin und als Hämoglobin-Addukt nach einem nicht näher beschriebenen Unfall. Der Methämoglobingehalt im Blut stieg bis zum Messpunkt drei Stunden nach dem Unfall auf 43,9 % an. Die p-Chloranilin-Konzentration im Urin erreichte 30 Minuten nach dem Unfall ihren Höchstwert und fiel dann stetig ab. Nach drei Tagen konnte kein p-Chloranilin mehr nachgewiesen werden. Die Konzentration von p-Chloranilin im Blut, das aus dem Hämoglobin-Addukt freigesetzt wurde, baute sich über die ersten drei Stunden nach dem Unfall auf und wurde deutlich langsamer eliminiert, sodass es noch eine Woche nach dem Unfall im Blut nachweisbar war (siehe Tabelle 1; Lewalter und Korallus 1985). Auch wenn der Unfall eine Mischexposition aus Anilin und p-Chloranilin darstellte, ist davon auszugehen, dass p-Chloranilin wegen des höheren Hämoglobin-Bindungsindex einen größeren Anteil an der Methämoglobinbildung hatte. In einem anderen Fall kontaminierte sich ein Arbeiter beim Umgang mit p-Chloranilin-haltigem Abfall und wies einen Methämoglobingehalt von 69 % auf. In seinem Urin wurde p-Chloranilin identifiziert. Jedoch wurde die Analytik des Urins auf p-Chloranilin nur qualitativ durchgeführt (Pizon et al. 2009). Ein Arbeiter, der bei Reinigungsarbeiten gegen p-Chloranilin dermal und möglicherweise wegen einer defekten Atemmaske auch inhalativ exponiert war, hatte einen Methämoglobingehalt von 42,8 %. Ein Biomonitoring auf die Metaboliten von p-Chloranilin in Blut und Urin wurde nicht durchgeführt (Messmer et al. 2015). Nach Hautkontakt mit p-Chloranilin und einsetzender Zyanose wurden bei einem anderen Arbeiter ein Methämoglobingehalt von 18 % und 161 µg p-Chloranilin/l Blut aus dem Hämoglobin-Addukt gemessen (siehe Tabelle 1; Herber 2024).

**Tab. 1:** Konzentrationen von Methämoglobin im Blut und p-Chloranilin im Urin bzw. im Blut (aus Hämoglobin-Addukt) nach Unfällen

Zeit nach Unfall	Met-Hb [%]	p-Chloranilin im Urin [µg/g Kreatinin]	p-Chloranilin als Hb-Addukt [µg/l Blut]	Literatur
30 min	36,2*	1500	100	Lewalter und Korallus 1985
3 h	43,9*	500	300	
7 h	15,9*	200	200	
16 h	1,2*	50	100	
3 d	0,3*	< 10	100	
7 d	1,0*	< 10	50	
12 d	0,9*	< 10	< 10	
–	18	–	161	Herber 2024

^*^ zusätzliche Anilin-Exposition

d: Tage; h: Stunden; Hb: Hämoglobin; Met-Hb: Methämoglobin; min: Minuten

## Auswahl der Indikatoren

Eine Belastung mit p-Chloranilin kann sowohl im Urin als auch im Blut nachgewiesen werden. Im Urin wird im Rahmen der Probenvorbereitung typischerweise eine Hydrolyse durchgeführt, bei der Phase-II-Konjugate des p-Chloranilins in freies p-Chloranilin umgewandelt werden. Auf diesem Wege wird sowohl freies als auch gebundenes p-Chloranilin detektiert. Eine Unterscheidung zwischen freiem und gebundenem p-Chloranilin ist nach der Hydrolyse nicht mehr möglich.

Außerdem können Hämoglobin-Addukte von p-Chloranilin bestimmt werden. Dieser Parameter beruht darauf, dass aromatische Amine in reaktive Nitroso-Spezies umgewandelt werden und dann mit einer Cystein-Thiolgruppe des Hämoglobins reagieren. Die so entstandenen Addukte sind über die gesamte Lebenszeit des Erythrozyten (ca. 120 Tage) stabil, können aber *in vitro* unter milden Bedingungen hydrolysiert werden.

Des Weiteren kann als biologischer Effektparameter das Methämoglobin bestimmt werden. Während ein gesunder Mensch nur ca. 1 % Methämoglobin aufweist (Mansouri und Lurie 1993), steigt dieser Wert bei Einwirkung von p-Chlor­anilin stark an. So wurden nach Arbeitsunfällen mit p-Chloranilin Methämoglobingehalte von 69 % nach inhalativer Exposition (Pizon et al. 2009) oder 42,8 % nach vermuteter dermaler Exposition beobachtet (Messmer et al. 2015). Als Biomarker weist Methämoglobin den Nachteil einer geringen Spezifität für p-Chloranilin auf. Die Ursache hierfür ist, dass sowohl aromatische Amine als auch diverse andere Stoffe, wie Nitrite und Chlorate, eine Methämoglobinbildung hervorrufen können.

## Untersuchungsmethoden

Meistens wird p-Chloranilin im Urin nach einer Hydrolyse gaschromatographisch bestimmt. Die im deutschsprachigen Raum wahrscheinlich am häufigsten angewandte Methode ist die Kapillargaschromatographie mit massenselektiver Detektion im Einzelionen-Monitoring-Modus (GC-MSD im SIM-Modus; Weiss und Angerer 2002). Eine ältere Methode unter Verwendung einer Gaschromatographie mit Elektroneneinfangdetektor (GC-ECD) wurde von Riffelmann et al. (1995) veröffentlicht. Zudem sind flüssigchromatographische Methoden mit UV- (Below et al. 2004; Jones et al. 2007) oder EC-Detektion (Lores et al. 1980) und in neueren Arbeiten auch mit massenselektivem Detektor bekannt (Chinthakindi und Kannan 2021).

Die Bestimmung von p-Chloranilin, das aus dem Hämoglobin-Addukt hydrolysiert wird, erfolgt in einer DFG-Methode mittels GC-MS mit negativer chemischer Ionisation (Lewalter et al. 2000).

## Hintergrundbelastung

In einer Studie von Riffelmann et al. (1995) wurde die Belastung mit verschiedenen aromatischen Aminen, darunter p-Chloranilin, von potentiell exponierten Arbeitern und einer Kontrollgruppe miteinander verglichen. Dafür wurde sowohl die Konzentration von p-Chloranilin im Urin als auch die Konzentration von p-Chloranilin im Blut freigesetzt aus dem Hämoglobin-Addukt bestimmt. Die Kontrollgruppe bestand aus 16 Männern (8 Raucher, 8 Nichtraucher). Im Folgenden werden nur die Messungen von p-Chloranilin im Urin berichtet: Bei den acht Nichtrauchern konnte kein p-Chloranilin nachgewiesen werden, wohingegen bei den acht Rauchern bei weniger als der Hälfte p-Chloranilin nachgewiesen werden konnte. Die maximale Konzentration lag bei 0,8 µg p-Chloranilin/l Urin.

Weiß und Angerer untersuchten den Urin von 20 Personen (Geschlecht unbekannt) auf p-Chloranilin. Bei einer Nachweisgrenze von 0,05 µg/l Urin betrug die Detektionsrate 90 %. Der Median lag bei 0,11 µg/l und das 95. Perzentil bei 0,57 µg p-Chloranilin/l Urin. Der Maximalwert war mit 1,1 µg p-Chloranilin/l Urin fast doppelt so hoch wie das 95. Perzentil (Weiss und Angerer 2002).

Weiß untersuchte in seiner Dissertation die Belastung von 197 Probanden (Geschlecht unbekannt) aus der Allgemeinbevölkerung mit verschiedenen aromatischen Aminen, darunter auch p-Chloranilin. Bei einer Nachweisgrenze von 0,05 µg/l Urin lag die Detektionsrate bei 85,8 %. Der Median betrug 0,185 µg/l und das 95. Perzentil 1,1 µg p-Chloranilin/l Urin (Weiß 2005).

Die bisher umfangreichste Bevölkerungsstudie zur Belastung mit aromatischen Aminen führten Kütting et al. (2009) an 911 Männern und Frauen (15–84 Jahre) sowie 93 Kindern (< 15 Jahre) aus Bayern durch. Hier lag der Median bei 0,03 µg p-Chloranilin/l Urin und damit unterhalb der Bestimmungsgrenze. Das 95. Perzentil lag bei 0,93 µg p-Chloranilin/l Urin. Das Gesamt-Kollektiv wurde in Raucher (n = 145) und Nichtraucher (n = 856) eingeteilt, um den Einfluss des Raucherstatus auf die p-Chloranilin-Belastung zu untersuchen. Auch für die beiden Subkollektive lag der Median unterhalb der Bestimmungsgrenze. Sowohl das 95. Perzentil als auch der Maximalwert mit 0,61 bzw. 3,02 µg p-Chloranilin/l Urin waren bei den Rauchern kleiner als bei den Nichtrauchern. Dort betrugen das 95. Perzentil 0,98 und das Maximum 42,13 µg p-Chloranilin/l Urin (Kütting et al. 2009). Die Diskrepanz zwischen den Werten ist mit hoher Wahrscheinlichkeit einer Verzerrung aufgrund der stark unterschiedlichen Kollektivgrößen geschuldet. Ein Einfluss des Raucherstatus ist jedenfalls nicht ersichtlich.

In einer Studie an 15 US-amerikanischen Probanden (7 Männer und 8 Frauen, 10 Asiaten und 5 Kaukasier; 213 Proben (10–16 pro Proband)) lag bei einer Nachweisgrenze von 0,05 µg/l die Detektionsrate bei 67,7 %. Der Median betrug 0,085 µg/l Urin bzw. 0,051 µg p-Chloranilin/g Kreatinin und das 95. Perzentil 1,326 µg/l Urin bzw. 1,238 µg p-Chloranilin/g Kreatinin. Das Maximum betrug 3,84 µg/l Urin bzw. 3,05 µg p-Chloranilin/g Kreatinin (Chinthakindi und Kannan 2022).

Die Daten zur Hintergrundbelastung sind in Tabelle 2 dargestellt.

**Tab. 2:** p-Chloranilin-Ausscheidung im Urin der beruflich nicht gegenüber p-Chloranilin exponierten Allgemeinbevölkerung.

**Kollektiv**	**% > NWG/BG**	**p-Chloranilin im Urin [µg/l Urin] (µg/g Kreatinin)**					**Literatur**
		**Mittelwert**	**Median**	**95. Perzentil**	**Bereich**	**NWG/BG**	
**Deutschland**														
8 Raucher	–	0,1	< NWG	–	< NWG–0,8	1/–	Riffelmann et al. 1995
8 Nichtraucher	–	< NWG	< NWG	< NWG	< NWG	1/–	
197	85,8	–	0,185	1,101	< NWG–39,1	0,05/–	Weiß 2005
98, städt. Bevölkerung	81,6	–	0,184	1,219	< NWG–2,562	0,05/–	
99, ländl. Bevölkerung	89,9	–	0,186	1,015	< NWG–39,1	0,05/–	
20	90	–	0,11	0,57	< NWG–1,1	0,05/–	Weiss und Angerer 2002
1004	38,2	0,31	< BG	0,93	< BG–42,13	–/0,03	Kütting et al. 2009
145 Raucher	34,5	0,15	< BG	0,61	< BG–3,02	–/0,03	
856 Nichtraucher	38,5	0,33	< BG	0,98	< BG–42,13	–/0,03	
**USA**														
n = 15, 213 Proben (→ mehrere Proben pro Person)	67,7	0,25 (0,17)	0,085 (0,051)	1,326 (1,238)	< NWG–3,84	0,05/–	Chinthakindi und Kannan 2022

BG: Bestimmungsgrenze; NWG: Nachweisgrenze

## Evaluierung

Da sich p-Chloranilin im Tierversuch als eindeutig kanzerogen erwies und in Kanzerogenitätskategorie 2 eingestuft wurde, wird kein Biologischer Arbeitsstoff-Toleranzwert (BAT-Wert) abgeleitet. Die Datenlage reicht nicht aus, um einen Biologischen Leitwert (BLW) abzuleiten. Eine Ableitung von Expositionsäquivalenten für krebserzeugende Arbeitsstoffe (EKA) ist aufgrund der fehlenden Daten sowohl für Blut als auch Urin ebenfalls nicht möglich. Die Datenlage zur Hintergrundbelastung von p-Chloranilin im Urin hingegen ist ausreichend, um einen Biologischen Arbeitsstoff-Referenzwert (BAR) abzuleiten. Am bedeutendsten für die Ableitung des BAR ist die Feldstudie an 1004 Personen aus Bayern – hier wurde ein 95. Perzentil von 0,93 µg p-Chloranilin/l Urin berichtet (Kütting et al. 2009). Die 95. Perzentile von 0,57 µg/l (Weiss und Angerer 2002), 1,1 µg/l (Weiß 2005) und 1,326 µg/l (Chinthakindi und Kannan 2022) aus den anderen Studien lagen in derselben Größenordnung und sind daher als Bestätigung des Ergebnisses von Kütting et al. (2009) anzusehen. Daher wird ein

**BAR von 1 µg p-Chloranilin (nach Hydrolyse)/l Urin**

festgelegt. Die Probenahme sollte am Expositions- bzw. Schichtende erfolgen. Der bei Riffelmann et al. (1995) diskutierte mögliche Einfluss des Raucherstatus auf die p-Chloranilin-Belastung konnte in den späteren Studien nicht beobachtet werden, sodass nicht von einem relevanten Einfluss des Rauchens auf die Ausscheidung von p-Chloranilin im Urin auszugehen ist. Da bis auf eine Studie alle anderen Studien die p-Chloranilin-Messwerte mit Volumenbezug berichtet haben, wird auch beim BAR der Volumen- und nicht der Kreatinin-Bezug gewählt.

## Interpretation

Der BAR bezieht sich auf normal konzentrierten Urin, bei dem der Kreatiningehalt im Bereich von 0,3–3 g/l liegt. In der Regel empfiehlt sich bei Urinproben außerhalb der oben genannten Grenzen die Wiederholung der Messung beim normal hydrierten Probanden (Bader und Ochsmann 2010).
